# Cortical Structure Alterations in Young People With Mild Internet Gaming Disorder

**DOI:** 10.1111/adb.70154

**Published:** 2026-04-14

**Authors:** Bohui Mei, Longyao Ma, Qiuying Tao, Jinghan Dang, Jieping Sun, Mengzhe Zhang, Jingliang Cheng, Yong Zhang

**Affiliations:** ^1^ Department of Magnetic Resonance Imaging, the First Affiliated Hospital of Zhengzhou University Zhengzhou China

**Keywords:** Internet gaming disorder, structural magnetic resonance imaging, surface‐based morphometry, voxel‐based morphometry

## Abstract

This study aims to explore alterations in cortical structure in young individuals with early Internet gaming disorder (IGD) and to provide novel insights for early detection and intervention of disease treatment. We investigated the brain structural magnetic resonance imaging data of 64 individuals with IGD and 47 healthy controls (HCs). The grey matter volume, cortical thickness, and cortical complexity of the two groups were compared separately, and the correlations between the brain regions with structural changes and clinical characteristics in IGD patients were analyzed. Compared to HCs, the IGD group showed no significant GMV differences. However, they exhibited a distinct pattern of right‐hemisphere dominant cortical thickening in regions spanning the default mode, dorsal attention, and visual networks (e.g., superior frontal gyrus [SFG], posterior cingulate and lateral occipital gyrus), concurrent with reduced cortical complexity in prefrontal and occipito‐parietal areas. Our study suggests that long‐term game stimulation is consistent with adaptive resource reallocation. Adolescents with mild IGD demonstrate a unique thickening‐complexity decoupling pattern, where cortical thickening in game‐related networks coexists with reduced complexity while GMV remains stable. This pattern could reflect a compensatory neuroadaptation phase, highlighting both a critical window for early intervention and specific neural targets for future therapeutic strategies.

## Graphical abstract

This study employed a voxel‐based and surface‐based morphometric method to discover that adolescents with mild Internet Gaming Disorder (IGD; IAT scores 50–80) might be characterized by a thickening‐complexity decoupling in right‐hemisphere networks, which may represent a compensatory neuroadaptation phase. Adolescents with mild IGD show right‐hemisphere cortical thickening (orange) in default mode, dorsal attention, and visual networks, alongside reduced cortical complexity (purple). Grey matter volume remains unchanged.

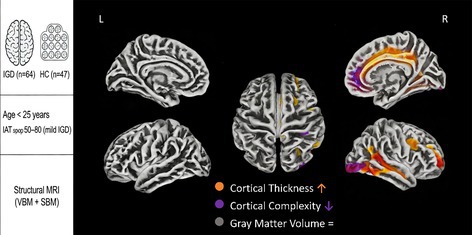

## 1 Introduction

The prevalence of Internet has led to a rising number of youths developing an addiction to online games, prompting considerable alarm regarding the escalating issue of Internet addiction [1]. Internet gaming disorder (IGD) is a form of excessive gaming behaviour characterized by impulsivity and a deficiency in self‐regulation. Its prevalence ranges from 0.6% to 19.9%, contingent upon the population and region [2, 3]. Multiple researches have proven that IGD can result in adverse effects such as vision impairment, sleep deprivation, difficulties in interpersonal communication, depression and anxiety [4, 5]. The impact on adolescents is particularly alarming, as their immature mental development renders them more susceptible to suicidal tendencies and criminal behaviour due to Internet game addiction [6], thereby imposing a significant burden on society.

Given that these behaviours are rooted in modifications in corresponding brain functions [7], it is necessary to better understand the neural changes associated with IGD. The leading hypothesis on the pathological basis of IGD suggests it is primarily characterized by excessive activation of the ventral striatum‐centred reward system and dopaminergic pathways, which drive gaming cravings [8]; impaired functioning of the anterior cingulate cortex (ACC), orbital frontal cortex (OFC) and other prefrontal regions responsible for cognitive control, leading to inadequate behavioural inhibition [9]; and morphological changes, such as grey matter volume (GMV) and cortical thickness (CT), interact with abnormal dynamic patterns of neural activity [10, 11]. Despite the proposal of several interventions for IGD based on extant studies [12, 13], further testing is required to ascertain the universality, pertinence and effectiveness. The prevailing paradigm for treatment continues to emphasize the early detection and intervention.

Structural magnetic resonance imaging (MRI) studies primarily encompass voxel‐based morphometry (VBM) [14] and surface‐based morphometry (SBM) [15], both of which utilize T1‐weighted MRI to estimate cortical morphology and thus objectively delineate the structural changes of the central nervous system. Structural indicators are relatively stable over time, which lends them diagnostic value [16]. VBM is predominantly used to estimate GMV [14]. Despite the plethora of studies conducted on GMV in IGD, the findings are frequently inconsistent or even contradictory. For example, a study demonstrated that the GMV in bilateral caudate nucleus and left nucleus accumbens (NAc) in IGD was increased compared with healthy controls (HCs) [8]; there are also VBM studies that showed that the volume of the ACC, inferior frontal gyrus (IFG), and insula decreased in IGD patients, while only the angular gyrus increased [17]. In addition, there is a theory proposed that games could strengthen the training of relevant skills, and the subjects showed a significant increase in grey matter (GM) in the hippocampal formation, PFC and bilateral cerebellum after game training [18]. The heterogeneity of these results may be attributable to the fact that GM loss is uncommon in adolescents or young adults with less severe disease [19], and simple VBM analysis may fail to thoroughly reveal subtle or complex changes in brain structure [20].

It has been suggested that measures of CT reflect earlier neurodevelopment and are more sensitive than GMV in suggesting cortical abnormalities [21]. SBM can measure various cortical features including volume, CT, gyrification index (GI), fractal dimension (FD), and sulcal depth (SD), with the latter three reflecting cortex complexity [22]. Existing SBM studies on IGD are scarce, with most finding extensive cortical thinning in ACC, OFC, supplementary motor area (SMA), posterior cingulate cortex (PCC), and inferior parietal lobular (IPG) [23, 24]. A fine‐grained analysis of human brain structure in relation to online gaming has also been conducted, revealing a strong positive correlation between the thickness of PFC and the duration of online gaming, with no brain region showing cortical thinning [25]. Moreover, there is a paucity of research that has investigated more subtle changes in cortical complexity in IGD. A recent review comparing two methods of structural MRI suggests that each has its own strengths and should be used concurrently to provide complementary information to improve the accuracy of detecting cortical morphological changes [26].

In summary, the classical theory suggests that IGD leads to brain atrophy and impaired cognitive, reward, and control functions, while some studies have suggested that online games can lead to gains in cognition and game‐related skills, such as eye movement and fine motor control of the hand. Therefore, the present study focused on young patients with mild IGD, used VBM to analyze cortical volume and SBM to analyze CT and complexity. This comparative study was conducted with a view to completing a thorough understanding of the neuroimaging mechanism of early IGD from the morphological perspective. We hypothesize that indicators of cortical complexity are more sensitive than CT and that for young patients with mild IGD, there exists a relatively stable phase of structural reorganization with simplified cortical complexity and a thickened cortex.

## 2 Methods

### 2.1 Subjects

Initially, 113 participants were recruited and assessed through outpatient clinics and online recruitment: the IGD group (*n* = 66, 54 males) and the control group (*n* = 47, 33 males). These subjects were screened using the Internet Addiction Test Questionnaire (IAT) and were critically assessed by two neurologists on the day of the experiment based on the nine‐item DSM‐5 criteria. The inclusion criteria for the IGD group, as recommended by psychiatrists, were as follows: (1) meeting the DSM‐V diagnostic criteria for IGD (at least five of the nine criteria); (2) IAT scores range from 50–80, with predominant Internet usage for gaming; (3) age under 25 years; (4) no history of substance abuse or dependence aside from IGD; and (5) right‐handedness. Exclusion criteria for both IGD and HC groups included the following: (1) contraindications for MRI scanning, such as metal implants, pacemakers or claustrophobia; (2) current use of psychotropic or other drugs; (3) presence of psychiatric, neurological or hereditary familial diseases; (4) severe traumatic brain injury or organic brain disease; and (5) significant cognitive impairment assessed using the Montreal Cognitive Assessment Scale (MoCA). Following MRI data quality control, two male subjects in the IGD group were excluded due to excessive head motion and noise artefacts, which resulted in a CAT12 quality rating below ‘C’ [27]. Therefore, the final analyzable sample comprised 64 IGD subjects (52 males) and all 47 HCs (33 males), with no exclusions from the HC group based on imaging quality.

Considering that IGD often has psychiatric comorbidities [28], we also collected the scores of the Hamilton Anxiety Scale (HAMA), the 17‐item Hamilton Depression Rating Scale (HAMD‐17) and the Yale‐Brown Obsessive Compulsive Scale (Y‐BOCS) of IGD subjects to assess clinical features.

This study was approved by the Medical Ethics Committee of the First Affiliated Hospital of Zhengzhou University and complied with the ethical guidelines of the World Medical Association (Declaration of Helsinki). All participants provided written informed consent prior to participation in the study.

### 2.2 Image Acquisition

All MRI images were acquired using a 3.0T MRI scanner (MAGNETOM Prisma, SIEMENS, Germany) equipped with a 64‐channel receive array head coil. Subjects were instructed to keep their eyes closed and remain awake throughout the scan. Foam fillers and earplugs were employed to minimize head movement and scanner noise. High‐resolution T1‐weighted volumetric 3D images were acquired using a fast acquisition of gradient echo sequences. The acquisition parameters are as follows: repetition time/echo time (TR/TE) = 2300/2.32 ms, field of view (FOV) = 240 × 240 mm^2^, slices = 176, and voxel size = 0.9 × 0.9 × 0.9 mm^3^.

### 2.3 Data Processing

The structural images analyses were carried out using the Computational Anatomy Toolbox (CAT12.6 (r1450); http://www.neuro.uni‐jena.de/cat/), which integrates with the Statistical Parametric Mapping 12 (SPM12 (7219)) on the Matlab 2018a platform (MathWorks, Natick, MA, USA). The T1 images of all participants were visually inspected, and the origin was manually reset at the anterior–posterior commissure (AC‐PC) to ensure the accuracy of subsequent registration.

#### 2.3.1 VBM Preprocessing Steps

T1 images of all subjects were first segmented into GM, white matter (WM), and cerebrospinal fluid (CSF). The GM density map affine registration to the stereotactic Montreal Neurological Institute (MNI) space, high‐dimensional Diffeomorphic Anatomy Registration Through Exponentiated Lie (DARTEL) normalization and nonlinear modulation using the Jacobian determinants derived from the normalization process. Then, we used 8‐mm full width at half maximum (FWHM) Gaussian kernels to smooth the modulated GM images to reduce the alignment bias, improve the data signal‐to‐noise ratio, and make the images more consistent with the Gaussian distribution for sample statistics.

#### 2.3.2 SBM Preprocessing Steps

In a manner analogous to VBM, SBM first performs tissue segmentation. The cortical surface was reconstructed to construct accurate GM‐WM matter boundaries. Topological corrections were applied to ensure surface continuity and anatomical accuracy. The reconstructed surfaces were then mapped to a spherical coordinate system and nonlinearly registered to the fsaverage standard template using curvature‐based alignment algorithms to improve intersubject comparability. CT was defined as the shortest distance between the GM/WM boundary and the GM/CSF boundary at each vertex of the reconstructed surface. The GI, FD, and SD were subsequently extracted from the central surfaces of both hemispheres using the “Surface tool” function in Cat12. Because SBM employs two‐dimensional spatial normalization, its registration accuracy should be higher than that of VBM for statistical analysis of cortical morphometry [29]. The CT was smoothed using a 15‐mm FWHM Gaussian kernel, while a 25‐mm FWHM Gaussian kernel was employed to smooth the GI, FD, and SD.

The image quality score (IQR) was automatically generated by CAT12 during data processing, with data exhibiting IQR < 75% being excluded, and the total intracranial volume (TIV) was obtained and used as a covariate. All procedures were performed in accordance with the guidelines provided in the CAT12 software manual.

### 2.4 Statistical Analysis

Initially, all data normality was assessed using the Shapiro–Wilk test. Two‐sample *t*‐tests and Mann–Whitney *U* tests were applied to quantify group differences for normally and nonnormally distributed data, respectively. Chi‐square tests were conducted to analyze the sex ratio differences between groups. The threshold for statistical significance was set at *p* < 0.05. Normally distributed data are reported as means ± standard deviations (SD), while nonnormally distributed data are presented as medians (lower and upper quartiles).

Two‐sample *t*‐tests were employed to investigate the alterations in structural parameters, including cortical volume, thickness, and complexity, between the IGD and HC groups. In order to reduce the false discovery rate, family‐wise error correction was applied to correct for multiple comparisons in all analyses (cluster‐level FWE corrected at *p* < 0.05, cluster extent > 100 voxels). Of note, both age and sex were considered as covariates in this procedure, whereas TIV was entered just as a covariate in the GMV analysis [30]. For all significant intergroup differences identified, we calculated effect sizes using Cohen's d values alongside corresponding 95% confidence intervals to further validate the reliability of the findings [31]. Finally, CAT 12 was used to derive the mean values of each index within significant clusters, and Spearman's correlation coefficient was employed to evaluate the correlations between morphological changes (GMV, CT, GI, FD and sulci depth) and clinical characteristics in the IGD group (*p* < 0.05; two‐tailed) to explore potential associations.

## 3 Results

### 3.1 Demographics

The clinical demographics of all participants are presented in Table 1. There were no significant differences in age, sex, and TIV between the two groups. Compared with the control group, the IGD group had a significantly higher score in IAT (*p* < 0.001).

**TABLE 1:** The demographic and clinical data of all participants.

	IGD (*n* = 64)	HC (*n* = 47)	*p*
**Age (years)**	14.625 (±1.95)	15 (±4.26)	0.124^a^
**Sex (male/female)**	52/12	33/14	0.177^b^
**TIV**	1505.64(1319.43, 1691.85)	1523.91(1299.76, 1748.06)	0.779^c^
**IAT**	64.46 (±8.16)	32.25 (±2.06)	< 0.001^a^
**HAMA**	12 (6, 19)	—	
**HAMD‐17**	21 (11, 31.25)	—	
**Y‐B**	10 (5, 19)	—	

*Note:* Data are presented as mean ± standard deviation, median (lower quartile and upper quartile) for continuous variables, or frequencies for categorical ones.

Abbreviations: HAMA, Hamilton Anxiety Scale; HAMD‐17, 17‐item Hamilton Depression Rating Scale; HC, healthy control; IAT, Internet Addiction Test Questionnaire; IGD, Internet gaming disorder; TIV, total intracranial volume; Y‐BOCS, Yale‐Brown Obsessive Compulsive Scale.

^a^ *p* values were obtained by two‐sample *t*‐test.

^b^ *p* value for the gender difference was obtained by chi‐square test.

^c^ *p* value was obtained by Mann–Whitney *U* test.

### 3.2 Neuroimaging

**FIGURE 1:**
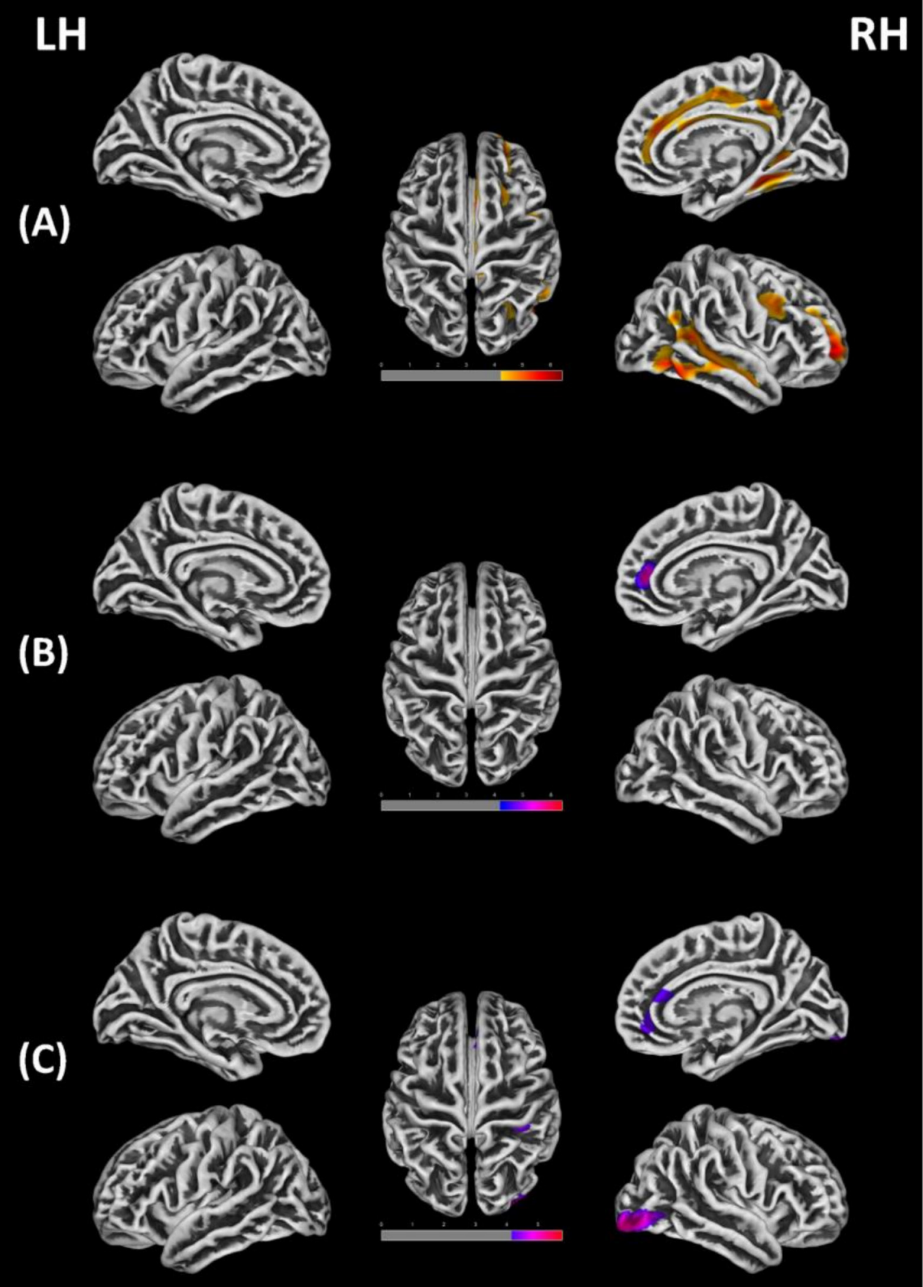
Cortical morphometric measures that are different between patients with Internet gaming disorder (IGD) compared to healthy controls (HC) (*p* < 0.05, family‐wise error correction). Yellow indicates brain regions with increased cortical thickness in the IGD group, while purple indicates brain regions with reduced cortical complexity in the IGD group. (A) The areas of increased cortical thickness in the patients with IGD compared to HC. (B) The areas of reduced fractal dimension in the patients with IGD compared to HC. (C) The areas of reduced sulcal depth in the patients with IGD compared to HC.

CT analysis showed that compared with HC, the IGD group had extensive thickening in the right hemisphere, including the right SFG, right caudal and rostral medial frontal gyrus (MFG), right posterior cingulate gyrus (PCC), right superior parietal gyrus (SPG), right middle temporal gyrus (MTG), right fusiform gyrus and right lingual gyrus (Table 2 and Figure 1A). There was no cortical thinning in any region.

**TABLE 2:** Brain regions where the cortical thickness was significantly increased in patients with Internet gaming disorder (IGD) compared to healthy controls (HC) (*p* < 0.05, family‐wise error correction).

			Peak MNI coordinates					
Hemisphere	Brain regions	Cluster sizes	X	Y	Z	*t*	Cohen's *d*	95% confidence interval
RH	Middle temporal	1340	67	−29	−10	5.8	1.12	[0.73,1.51]
	Inferior temporal							
	Bankssts							
	Inferior parietal							
	Superior temporal							
	Lateral occipital							
RH	Superior frontal	1061	14	36	22	5.2	1.00	[0.62,1.38]
	Posterior cingulate							
	Precuneus							
	Paracentral							
	Caudal anterior cingulate							
	Rostral anterior cingulate							
	Medial orbitofrontal							
	Isthmus cingulate							
RH	Fusiform	512	36	−41	−14	5.5	1.06	[0.67,1.45]
	Parahippocampal							
	Lingual							
	Inferior temporal							
RH	Caudal middle frontal	332	26	17	43	4.9	0.94	[0.57,1.31]
	Precentral							
	Rostral middle frontal							
	Parsopercularis							
RH	Rostral middle frontal	307	24	58	1	5.7	1.10	[0.71,1.49]
RH	Posterior cingulate	249	6	−26	28	5.1	0.98	[0.60,1.36]
	Caudal anterior cingulate							
	Isthmus cingulate							
RH	Superior parietal	153	29	−67	24	4.6	0.89	[0.52,1.26]
	Inferior parietal							
RH	Lingual	145	25	−54	2	5.1	0.98	[0.60,1.36]
	Isthmus cingulate							
	Precuneus							
RH	Superior frontal	124	26	16	44	4.7	0.91	[0.54,1.28]
	Caudal middle frontal							
RH	Inferior parietal	111	58	−54	−15	5	0.96	[0.58,1.34]

*Note:* Atlas labelling was performed according to the Desikan–Killiany atlas.

Abbreviations: LH, left hemisphere; MNI, Montreal Neurological Institute; RH, right hemisphere.

Cortical complexity analysis indicated a significant reduction of FD in the right SFG within the IGD group compared to HC (Table 3 and Figure 1B). Additionally, substantial decreases in sulcus depth were noted in the right medial orbitofrontal cortex (OFC), right lateral occipital gyrus, and right supramarginal gyrus for the IGD group (Table 3 and Figure 1C). However, no statistically significant differences in GI were observed between the groups, nor were there any increases in cortical complexity in any region.

**TABLE 3:** Brain regions where the cortical complexity was significantly reduced in patients with Internet gaming disorder (IGD) compared to healthy controls (HC) (*p* < 0.05, family‐wise error correction).

			Peak MNI coordinates					
Hemisphere	Brain regions	Cluster sizes	X	Y	Z	*t*	Cohen's *d*	95% confidence interval
**Fractal dimension**								
RH	Superior frontal	149	14	45	10	5.8	1.12	[0.73,1.51]
	Rostral anterior cingulate							
	Medial orbitofrontal							
**Sqrtsulc**								
RH	Lateral occipital	261	34	−92	−10	5.3	1.02	[0.64,1.40]
RH	Medial orbitofrontal	163	13	42	−2	4.4	0.85	[0.49,1.21]
	Rostral anterior cingulate							
	Caudal anterior cingulate							
	Superior frontal							
RH	Supramarginal	162	40	−36	39	4.7	0.91	[0.54,1.28]
	Postcentral							
	Superior parietal							

*Note:* Atlas labelling was performed according to the Desikan–Killiany atlas.

Abbreviations: LH, left hemisphere; MNI, Montreal Neurological Institute; RH, right hemisphere.

Notably, the GMV analysis showed that there was no statistically significant difference between IGD and HC groups.

### 3.3 Correlation Analysis

Spearman correlation analysis demonstrated a negative association between clinical indications of Internet addiction and cortical complexity (Figure S1), but not with CT. Specifically, IAT scores were negatively correlated with FD in the right SFG (*r* = −0.418, *p* = 0.002) and sulcus depth in the right lateral occipital gyrus (*r* = −0.355, *p* = 0.008), while HAMA scores were negatively correlated with sulcus depth in the right supramarginal gyrus (*r* = −0.286, *p* = 0.034). In addition, correlations among behavioural measures were assessed. Positive correlations were observed among depression, anxiety, and impulsivity (HAMD and HAMA, *r* = 0.758, *p* < 0.001; HAMD and Y‐B, *r* = 0.734, *p* < 0.001; HAMA and Y‐B, *r* = 0.778, *p* < 0.001), though none of them was significantly correlated with the severity of IGD. However, after Bonferroni correction, these correlation results were not significant. All exploratory analyses that did not pass post hoc validation are presented in the supplementary materials. These findings remain preliminary and require further validation in future studies.

## 4 Discussion

Using a combination of SBM and VBM methods, we systematically investigated the cortical morphological changes in young patients with mild IGD. The structural reorganization effect, characterized by cortical thickening and simplification, predominantly in the right default mode network (DMN), the dorsal attention network (DAN) and the visual network (VN) was clearly demonstrated in this mild cohort. These results challenge the general expectation of the traditional addiction brain model and may be the cost of neural optimization of game‐related skills, which is helpful for early diagnosis and timely intervention of IGD.

The PFC is a crucial node in brain architecture, extensively interconnected with other brain regions [32]. It has been pointed out that the PFC can rapidly integrate various types of information from other brain regions and issue regulatory commands, typically inhibitory [33], playing a significant role in fundamental brain functions such as behaviour execution, emotion processing, and cognitive regulation [34]. Our results suggest that young patients with mild IGD exhibit increased thickness and decreased complexity of PFC. The observed neural thickening may be attributable to prolonged gaming training. Previous studies have demonstrated that such training enhances working memory and decision‐making abilities, potentially linked to reactive glial cell proliferation or increased dendritic spine density in the relevant neural networks [35, 36]. In contrast, synapses in nongame‐related cognitive pathways (social cognitive processing) are selectively pruned, which could lead to simplified sulcal gyrus geometries [37, 38], impair the efficiency of transcerebral integration of information, and ultimately narrow related neural pathways [39]. Specifically, adolescents with IGD may have prolonged response time in the Stroop colour‐word task [40]. It should be noted that, given the cross‐sectional nature of our data, while it suggests a compensatory neuroadaptive phase, it does not definitively establish the existence of this phase. Longitudinal studies are still required to confirm the causal relationship between long‐term gaming behaviour and this structural reorganization, and to track its potential developmental trajectory. Similarly, the supramarginal gyrus [41], a core node of the DMN, and the lateral occipital gyrus [42], a key brain area of the VN, showed similar changes, both of which play an important role in the initiation and maintenance of online games. Reduced SD in the lateral occipital gyrus, which is responsible for visual processing, can reduce the cross‐gyrus information transfer hierarchy to enhance visual processing speed [43]. However, this relatively weakens visual information processing, which results in game players' visual attention bias, over‐sensitivity to game cues, and reduced ability to process the details of realistic scenes [44]. Players exhibit heightened efficiency in capturing game cues, yet diminished executive control, making them prone to impulsive behaviour that further intensifies cravings or recurrence. This could be a target of choice for clinical neuromodulation therapy, and its relatively superficial location greatly enhances the feasibility of the protocol.

Our results showed the supramarginal gyrus, a component of the inferior parietal gyrus (IPG), exhibited reduced SD. The IPG is mainly involved in somatosensory integration [45]. Cortical simplification impairs the accuracy of proprioceptive integration and weakens the granularity of neural decoding of somatic signals. This may explain why IGD patients ignore the physiological discomfort of prolonged gaming, as well as why patients' somatization symptoms aggravated during withdrawal [46]. This provides a new perspective for the recovery and withdrawal of IGD, and the supramarginal gyrus can be used as a therapeutic target for withdrawal symptoms to prevent relapse.

It has been shown that the CT of primary motor and somatosensory cortex is relatively high in groups in occupations with higher physically complex activities [47]. This could explain our findings that prolonged gaming experience enhances players' visual–spatial skills, cognitive decision‐making ability, and thickening of brain regions that are highly matched with the cognitive demands of gaming behaviour. The DMN (medial PFC, PCC and supramarginal gyrus) supports contextual memory and self‐projection [48], and the expansion of its core hubs supports game strategy optimization. The DAN (SFG and SPG) is responsible for spatial attention allocation [49]. Its thickening might reflect the neuroplasticity driven by the needs of spatial navigation in game, which greatly improves the spatial working memory capacity and joystick manipulation accuracy. The VN (lateral occipital gyrus, fusiform gyrus and lingual gyrus) process fast‐moving complex images [50], and the adaptive thickening may have occurred in response to the high‐frequency visual stimuli. The aforementioned factors may constitute the neurological basis for the game‐related skill gains that online games can facilitate. Therefore, we call for online games should not be denied, and the ability to produce desired brain changes should be explored. Future task‐based or resting‐state fMRI studies could clarify whether these structural changes correspond to enhanced game‐related attention, visual processing, or self‐referential functions.

Intriguingly, all brain regions with structural changes were concentrated in the right hemisphere, a region critical for social cognition, visuospatial attention, and memory functions [51]. It is now widely accepted that brain asymmetry is a fundamental element of the human sensory, cognitive and motor systems [52]. This finding emphasizes the early damage in cognitive and visual functions related to the right hemisphere of IGD patients. Nonetheless, it contrasts with common neuroimaging studies that found the left hemisphere showed greater intraregional connectivity in brain regions involved in fine motor coordination [53], particularly since the subjects in our study were right‐handed and the movement of the right arm is usually carried out by the left parietal lobe under visual guidance [54]. We suspect that the absence of restrictions on the types of online games played (some games mainly operated by the left hand) may have influenced our results [55]. Therefore, future research ought to examine the effects of different game types on neural changes to validate our findings.

Overall, our results are not in line with traditional addiction research, but support the hypothesis that there is a compensatory period of structural reorganization in young patients with mild IGD. The classic addiction theory posits that IGD patients exhibit a proclivity for brain atrophy, characterized by a general decrease in GMV and CT within the frontal‐striatal and frontal‐cingulate circuits [56, 57]. However, in our study, the GMV of the brain regions associated with game behaviour in adolescents with mild IGD was unchanged, and the CT was increased, whilst the cortical complexity was decreased. The generalisability of results from different studies can be affected by inclusion criteria, sample heterogeneity and sample size. The present study focused on IGD subjects younger than 25 years of age and with IAT scores between 50 and 80, when they may be in a clinical compensatory phase, with structural expansion compensating for functional load, and the increase in CT offset by a decrease in complexity. Because GMV is determined by both CT and cortical surface area, it ultimately manifests as unchanged [58]. GM loss is not easily observed in young individuals [59], and the estimation of CT and complexity has been proved to be more sensitive to detect subtle cortical differences in Parkinson's disease [21] and autism [60]. It is suggested that at this stage of the study, CT increase and cortical folding simplification reached a dynamic equilibrium to maintain the relative stability of GMV. This precise balance of structural reorganization may be a sign of the window period for early intervention, which can be used as an early warning of IGD through the complexity index. Neuroplasticity training during the compensatory phase may reverse the process of structural reorganization; if not treated in time, it will further develop into an atrophic decompensation phase with cortical thinning and extensive reduction in GMV, and treatment resistance will emerge.

Our study has several limitations. Firstly, we studied five morphological features using the CAT12 toolbox to analyze the morphological brain network in IGD. Future research should explore additional features like GM surface, mean curvature, and WM fibre tracts to enhance our understanding. GMV is determined by both thickness and surface area. GM reorganization is often accompanied by WM fibre remodelling. In the future, it is necessary to explore the changes of GM surface area and WM fibre bundles in IGD patients to further improve the overall results of brain structural changes in the mild compensatory stage. Secondly, the limited sample size constrains our conclusions. Because the diagnostic classification of behavioural addiction is still controversial, the IAT score criteria of patients with mild IGD included in this study were mainly determined based on clinical experience, and none exhibited cognitive impairment. In future research, expanding the number of participants and refining the severity of addiction is essential to ensure clinical reproducibility. Thirdly, it cannot be denied that, despite our efforts to restrict our study to participants whose primary Internet usage was online gaming, the majority also engaged in other online activities beyond gaming. It may be necessary to refine our experimental design in the future in conjunction with functional studies to focus solely on subjects with gaming‐specific neural reactions. Fourthly, this study did not limit the categories of online games. Future research should revisit the differentiation of online game kinds (such as competitive, strategic, business) to elucidate their unique effects on the nervous system. Fifthly, this is a case–control cross‐sectional study, which cannot definitively conclude the causal relationship between brain structural changes and excessive Internet gaming. Longitudinal investigations are necessary for a deeper understanding.

## 5 Conclusions

In conclusion, in young patients with mild IGD, under the long‐term stimulation of gaming behaviour, there is an increase in CT and a reduction in cortical complexity of game‐related brain regions. The essence of this thickening may suggest preferential allocation of neural resources, which is manifested as enhanced self‐projection of DMN, spatial navigation optimization of DAN, and dynamic processing enhancement of VN. The reduced cortical complexity indicates the decrease of cognitive flexibility and the loss of network balance, which is the cost of this adaptive reorganization. At this point, the structural changes have not yet affected GMV, suggesting a reversible stage of neuroadaptive changes, which provides a targeted basis for early precise intervention. Future research should focus on the detection of thickening‐complexity imbalance tipping point, with the aim of halting the progression of lesions into extensive pathological atrophy and achieving early diagnosis and timely intervention.

## Funding

This work was supported by the National Natural Science Foundation of China (81601467, 81871327, and 62106229) and Funding for Scientific Research and Innovation Team of the First Affiliated Hospital of Zhengzhou University (QNCXTD2023007).

## Ethics Statement

All procedures performed in studies involving human participants were in accordance with the ethical standards of the institutional and/or national research committee and with the 1964 Helsinki declaration and its later amendments or comparable ethical standards. This study was approved by the Medical Ethics Committee of the First Affiliated Hospital of Zhengzhou University.

## Conflicts of Interest

The authors declare no conflicts of interest.

## Supporting information


**FIGURE S1:** Correlations between morphometric measures in Internet gaming disorder (IGD) patients and behavioural outcomes (explore analysis). (A) The FD in the right SFG was negatively correlated with IAT scores. (B) The sulcus depth in the right lateral occipital gyrus was negatively correlated with IAT scores. (C) The sulcus depth in the right supramarginal gyrus was negatively correlated with HAMA scores. HAMA, Hamilton Anxiety Scale; IAT, Internet Addiction Test Questionnaire; SFG, superior frontal gyrus.

## Data Availability

The data that support the findings of this study are available from the corresponding author upon request. This includes the processed group‐level statistical maps, the extracted region‐of‐interest values, and the demographic data. Requests will be promptly evaluated based on a predefined data transfer agreement to ensure compliance with ethical guidelines. We are committed to supporting the scientific community and encourage interested researchers to contact us for collaboration.
