# Supplementary material for: Cortical Structure Alterations in Young People With Mild Internet Gaming Disorder
Source: Addict Biol. 2026 Apr 14;31(4):e70154. doi: 10.1111/adb.70154 (PMC13077548; doi:10.1111/adb.70154)
Supplement: Supplementary file 1 — FIGURE S1: Correlations between morphometric measures in Internet gaming disorder (IGD) patients and behavioural outcomes (explore analysis). (A) The FD in the right SFG was negatively correlated with IAT scores. (B) The sulcus depth in the right lateral occipital gyrus was negatively correlated with IAT scores. (C) The sulcus depth in the right supramarginal gyrus was negatively correlated with HAMA scores. HAMA, Hamilton Anxiety Scale; IAT, Internet Addiction Test Questionnaire; SFG, superior frontal gyrus. [file ADB-31-e70154-s001.docx]

***Supplementary Material***


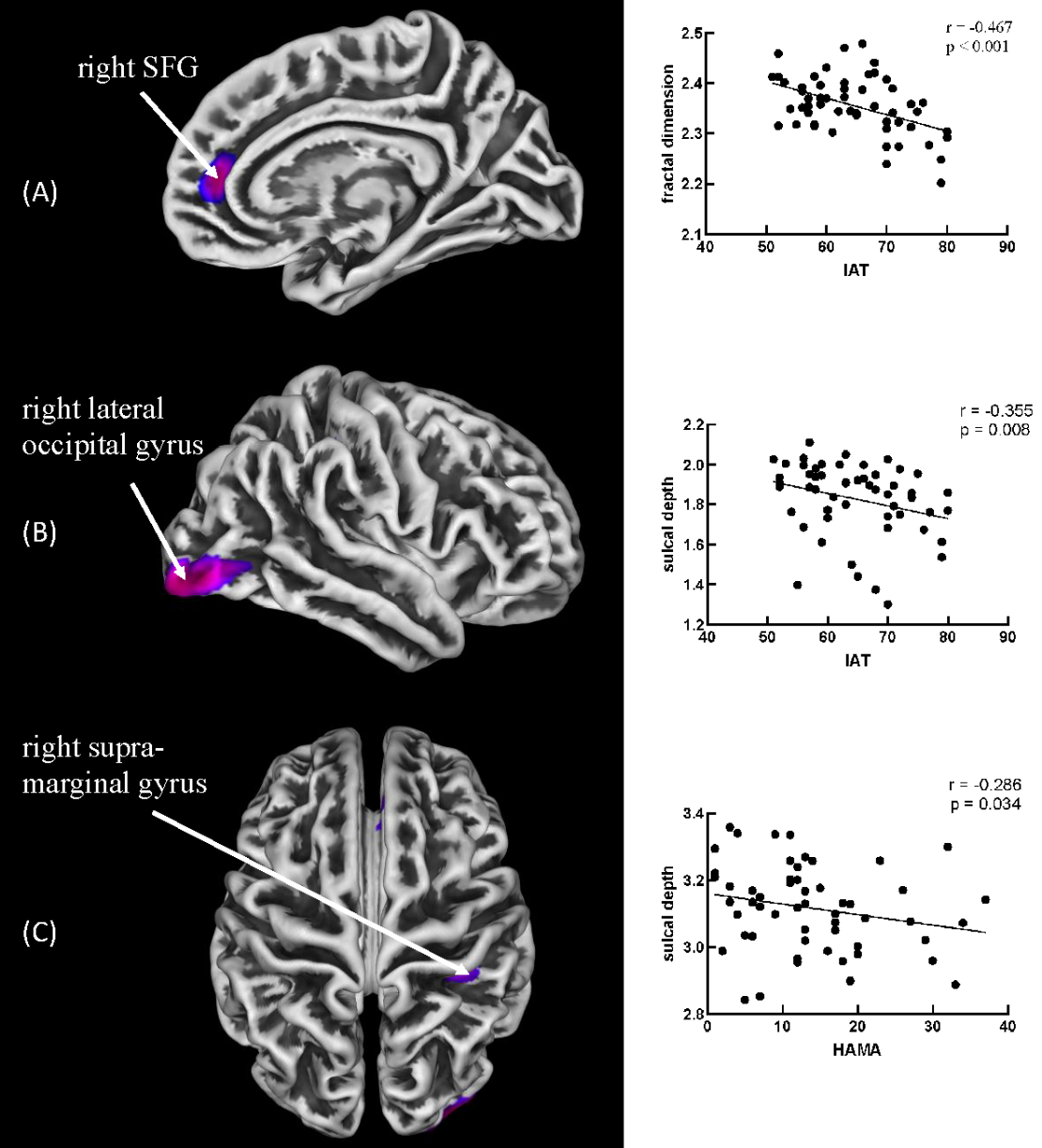


**FIGURE S1** Correlations between morphometric measures in Internet gaming disorder (IGD) patients and behavioral outcomes (explore analysis). (A) The FD in the right SFG was negatively correlated with IAT scores. (B)The sulcus depth in the right lateral occipital gyrus was negatively correlated with IAT scores. (C)The sulcus depth in the right supramarginal gyrus was negatively correlated with HAMA scores. Abbreviations: HAMA, Hamilton Anxiety Scale; IAT, Internet Addiction Test Questionnaire; SFG, superior frontal gyrus.
